# CASPT2 Potential Energy Curves for NO Dissociation in a Ruthenium Nitrosyl Complex

**DOI:** 10.3390/molecules25112613

**Published:** 2020-06-04

**Authors:** Francesco Talotta, Leticia González, Martial Boggio-Pasqua

**Affiliations:** 1 Institut de Chimie et Physique, UMR 8000 CNRS/Université Paris-Saclay, 91405 Orsay, France; francesco.talotta@universite-paris-saclay.fr; 2 Laboratoire de Chimie et Physique Quantiques, IRSAMC, CNRS/Université Toulouse 3, 118 route de Narbonne, 31062 Toulouse, France; 3 Institut für Theoretische Chemie, Fakultät für Chemie, Universität Wien, Währinger Strasse 17, 1090 Vienna, Austria; leticia.gonzalez@univie.ac.at; 4 Vienna Research Platform on Accelerating Photoreaction Discovery, Universität Wien, Währinger Strasse 17, 1090 Vienna, Austria

**Keywords:** photorelease, photoisomerization, photochromism, computational photochemistry, ab initio calculations

## Abstract

Ruthenium nitrosyl complexes are fascinating photoactive compounds showing complex photoreactivity, such as N→O linkage photoisomerism and NO photorelease. This dual photochemical behavior has been the subject of many experimental studies in order to optimize these systems for applications as photoswitches or therapeutic agents for NO delivery. However, despite recent experimental and computational studies along this line, the underlying photochemical mechanisms still need to be elucidated for a more efficient design of these systems. Here, we present a theoretical contribution based on the calculations of excited-state potential energy profiles for NO dissociation in the prototype *trans*-[RuCl(NO)(py)_4_]^2+^ complex at the complete active space second-order perturbation theory (CASPT2). The results point to a sequential two-step photon absorption photorelease mechanism coupled to partial photoisomerization to a side-on intermediate, in agreement with previous density functional theory calculations.

## 1. Introduction

Ruthenium nitrosyl complexes have attracted considerable interest over the last decades because of their multifunctional photoresponsive capability. Indeed, these complexes can display photochromism in the solid state [1,2,3,4,5,6,7,8,9,10,11,12,13,14,15] and be efficient agents for NO photorelease in solution [16,17,18,19,20,21,22,23,24,25,26,27,28,29,30]. Despite the numerous studies exploiting these two distinct photoreactivities (i.e., N→O linkage photoisomerization and photoinduced NO delivery), the underlying mechanisms are notoriously complex to unravel based on the available experimental observations [12,30].

**Figure 1:**
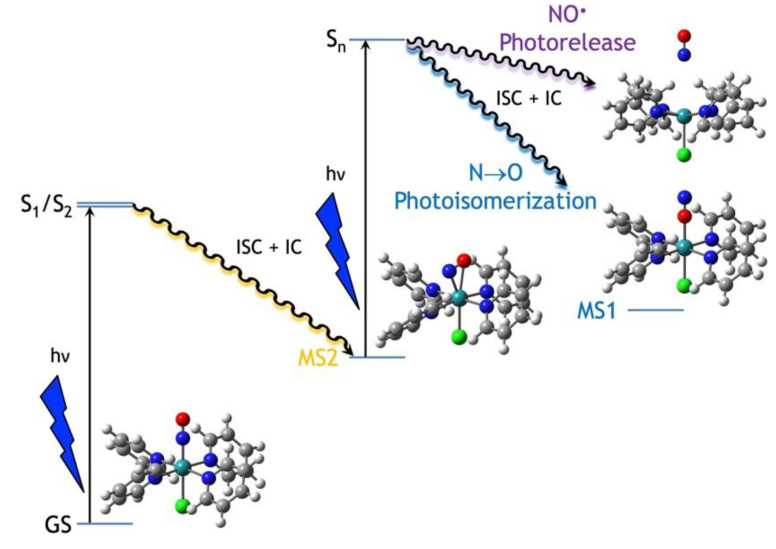
Scheme illustrating the competitive and intricated N→O linkage photoisomerization and NO^•^ photorelease mechanism in the *trans*-[RuCl(NO)(py)_4_]^2+^ complex. ISC: intersystem crossing. IC: internal conversion.

Computational photochemistry has proven to be a highly efficient tool to understand photo-induced molecular processes [31]. Although a vast majority of computational studies are dealing with organic photochemistry [32,33,34,35] due to the difficulty of computing photochemical pathways in metal complexes [36], recent computational investigations of photoisomerizable metal complexes have been published with the aim of understanding their photoswitching mechanisms [37,38,39,40,41,42,43,44,45]. Regarding ruthenium nitrosyl complexes, the N→O linkage photoisomerization mechanism in the *trans*-[RuCl(NO)(py)_4_]^2+^ (where py denotes a pyridine ligand) complex was investigated using density functional theory (DFT) and multi-state complete active space second-order perturbation theory (MS-CASPT2) to calculate the lowest singlet and triplet potential energy surfaces [43,44,45]. These studies revealed an activated and energetically uphill process in the lowest triplet excited state, which forbids a simple adiabatic mechanism in this electronic state, and suggested a complex sequential two-step photon absorption mechanism involving nonadiabatic processes (Figure 1). This mechanistic picture was later confirmed experimentally [12]. The proposed photoisomerization mechanism can be summarized as follows: upon blue-light absorption, the initial ground-state N-bonded (η^1^-N) species, GS, undergoes a partial isomerization to the η^2^-NO side-on metastable state MS2 following various internal conversions and intersystem crossings. This key intermediate then absorbs a second blue-light photon under continuous irradiation to produce the final N→O linkage isomer corresponding to the O-bonded (η^1^-O) isonitrosyl form, denoted MS1.

In addition to their ability to undergo linkage photoisomerizations, ruthenium nitrosyl complexes can also release nitric oxide upon light irradiation. The mechanism for NO^•^ photorelease is important, as this radical is involved in various physiological and pathological processes [46] and ruthenium nitrosyl complexes can be designed to promote NO^•^ photorelease, in particular using low-power light for biological and medicinal applications [24,26,29,47,48]. Experimental studies in the solid phase have shown that the photoproducts of both N→O linkage photoisomerization and NO^•^ photorelease could be observed, suggesting that weakly bound linkage isomers of nitric oxide are likely intermediates in the photolytic release of NO^•^ [19,22]. Very few theoretical studies have been devoted to NO^•^ photorelease. The initial stages of NO^•^ photorelease was investigated dynamically for the [Ru(PaPy_3_)(NO)]^2+^ complex (where PaPy3 = *N*,*N*′-bis(2-pyridylmethyl)amine-*N*-ethyl-2-pyridine-2-carboxamide) [49]. However, no excited-state intermediate could be identified as key transient species in the NO^•^ photorelease from these simulations. Later, a static DFT study investigating the lowest triplet potential energy surfaces of different ruthenium nitrosyl complexes identified a triplet η^2^-NO side-on structure denoted ^3^MS2, as the key intermediate for NO^•^ photorelease [50]. This finding suggested that partial photoisomerization to the η^2^-NO side-on MS2 metastable state was at least required for NO^•^ photorelease and that a sequential two-step photon absorption mechanism was involved like in the photoisomerization process (Figure 1). Soon after, a complete active space self-consistent field (CASSCF)-in-DFT embedding approach was used to explore the ground and excited electronic states of the *trans*-[RuCl(NO)(NH_3_)_4_]^2+^ complex along the Ru–NO stretching normal mode [51]. However, vibrational relaxation in the excited states was not taken into account and no information on the intermediates responsible for the NO^•^ photorelease was provided in that study.

The *trans*-[RuCl(NO)(py)_4_]^2+^ has often served as a prototype ruthenium nitrosyl system to study both N→O linkage photoisomerization and NO^•^ photorelease because of its capability to undergo both photoreactivities [9,10,11,12,52]. In this article, we present the results of accurate multireference ab initio CASPT2 calculations along NO^•^ photodissociation pathways in order to verify the validity of the DFT mechanistic picture previously reported [50] on this complex and to identify the most likely intermediate for NO^•^ photorelease. These results reveal further insight into how photorelease and photoisomerization are interconnected for this prototypical molecule.

## 2. Results and Discussion

### 2.1. Previous DFT Results of NO Photodissociation

**Figure 2:**
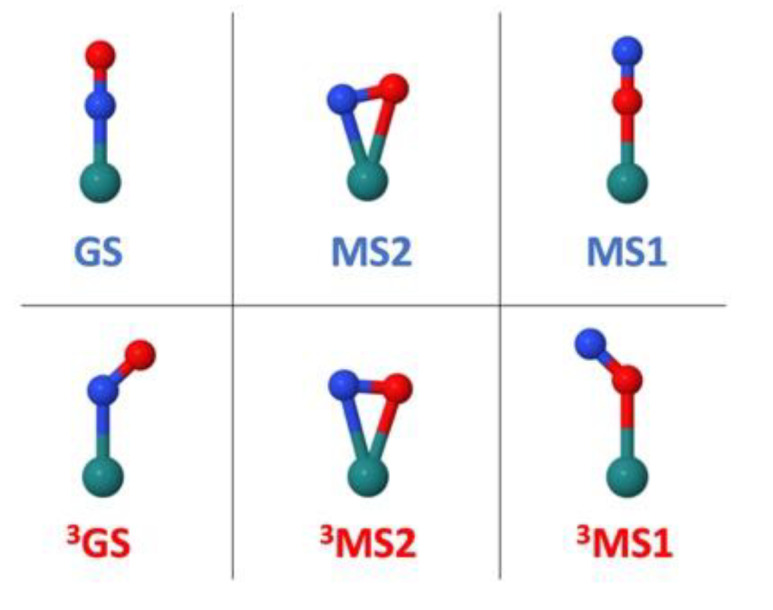
Illustration of the Ru–N–O geometrical arrangement in the singlet ground-state structures (GS, MS2, MS1) and in the triplet excited-states intermediates (^3^GS, ^3^MS2, ^3^MS1). Color code: Ru in cyan, N in blue and O in red.

The main mechanistic assumptions for the determination of the NO^•^ photorelease pathways is that i) this process occurs from the lowest triplet excited states, as suggested by the rapid population of these states after initial irradiation and ultrafast intersystem crossing (ISC) [45,49,53], and that ii) it involves initially a decoordination of the NO^•^ radical [19,22]. According to i), three triplet-state intermediates (Figure 2) were located on the lowest triplet potential energy surface at the DFT level [43,44,50]. The lowest energy intermediate is a triplet N-bonded (η^1^-N) species denoted ^3^GS, in which the Ru–N–O is bent, unlike the structure of the initial N-bonded GS that presents a collinear Ru–N–O alignment. Another triplet intermediate is the η^2^-NO side-on structure ^3^MS2 mentioned above. The third triplet state corresponds to an O-bonded (η^1^-O) species denoted ^3^MS1, in which the Ru–O–N is also bent, whereas it is collinear in the singlet MS1. All these intermediates are potential candidates for transient species in the NO^•^ photorelease mechanism. Thus, a straightforward computational strategy consists in calculating the dissociation energy of NO^•^ from these triplet excited-state species. If we write formally the chemical equations for the photorelease pathway—note that the triplet excited states ^3^GS, ^3^MS2 and ^3^MS1 all have a dominant MLCT character [43]—the ruthenium center can be written formally as Ru^III^ and one could postulate:

(1) $${\left[{\mathrm{Ru}}^{\mathrm{II}}{\mathrm{Cl}}^{-}\left({\mathrm{NO}}^{+}\right){\left(\mathrm{py}\right)}_{4}\right]}^{2+}\overset{h\nu }{\to }{\left[{\mathrm{Ru}}^{\mathrm{III}}{\mathrm{Cl}}^{-}\left({\mathrm{NO}}^{0}\right){\left(\mathrm{py}\right)}_{4}\right]}^{2+}\to {\left[{\mathrm{Ru}}^{\mathrm{III}}{\mathrm{Cl}}^{-}{\left(\mathrm{py}\right)}_{4}\right]}^{•2+}+\mathrm{NO}•$$

Although we know that it is not possible to assign such clear formal oxidation states to these species [54], it allows us to have a simple chemical representation of the expected fragments. From Equation (1), the Ru–NO photodissociation should produce two doublet states (radicals): one corresponding to a pentacoordinated ruthenium complex and one corresponding to nitric oxide.

DFT potential energy profiles for the NO^•^ photorelease pathways of the *trans*-[RuCl(NO)(py)_4_]^2+^ complex are reproduced in Figure 3 from Ref. [50]. They are based on the use of the B3LYP-D3 functional to calculate minimum energy paths along the NO dissociation from the three triplet excited states ^3^GS, ^3^MS2 and ^3^MS1. They show that the ^3^MS2 intermediate is the one that requires less energy to dissociate Ru–NO, in agreement with the fact that the ^3^MS2 intermediate is the highest energy triplet-state intermediate at this level of theory [43,50]. However, these energy profiles also reveal a wrong physical behavior at the asymptotic limit, as the energy is still substantially decreasing when the Ru–NO distance is increasing beyond 9 Å. According to Equation (1), the expected spin densities of the fragments produced should be exactly equal to 1. However, at the B3LYP-D3 level of theory, these spin densities are 1.2 and 0.8, for [Ru^III^Cl^−^(py)_4_]^•2+^ and NO^•^, respectively, indicating an artificial electron (charge) transfer between the NO^•^ and [Ru^III^Cl^−^(py)_4_]^•2+^ fragments. In addition, the NO^•^ radical has a doubly degenerate X^2^Π ground state, which cannot be physically described with a single-configuration representation of the DFT wavefunction. Thus, we report next CASPT2 potential energy curves for NO^•^ dissociation which address both issues.

### 2.2. CASPT2 Results of NO Photodissociation

The deficiencies of DFT can be overcome using CASPT2 to compute the Ru–NO photodissociation potential energy profiles starting from each intermediate. The results of the CASPT2 calculations are represented in Figure 4. The first obvious outcome of these calculations is that the lowest pair of singlet states S_0_/S_1_ and the lowest pair of triplet states T_1_/T_2_ all dissociate correctly to the same asymptote. Figure 4a,b shows that the complex dissociates to [RuCl(py)_4_]^•2+^ + NO^•^, with the pentacoordinated complex bearing its unpaired electron in the d_xz_ orbital of the Ru center, while NO^•^ has its unpaired electron either in a π_x_ or π_y_ orbital. Of course, the two doublet states of the dissociated fragments can be spin-coupled in a singlet or in a triplet state without changing the asymptotic limit. For the same reason, the next two pairs of singlet and triplet states are also degenerate between each other: S_2_/S_3_ and T_3_/T_4_ dissociate to an excited state of [RuCl(Py)_4_]^•2+^ + NO^•^ with the metal fragment bearing its unpaired electron in the d_xy_ orbital of the Ru center and NO^•^ in its doubly degenerate X^2^Π state. Finally, the third pair of singlet states S_4_/S_5_ is degenerate with T_5_/T_6_, where the complex is now having its unpaired electron in the d_yz_ orbital of the ruthenium.

It is important to note that these CASPT2 potential energy profiles are computed on the structures resulting from relaxed scans performed on the lowest triplet T_1_ state at the DFT level (see Computational Details). Given that B3LYP-D3 cannot describe the correct electronic structures at the asymptotic limit, we used CAM-B3LYP, which does not suffer from the artificial charge transfer problem between the dissociated fragments observed with B3LYP-D3, and thus provides the expected spin densities of 1.0 for the metal complex and the NO^•^ fragments and the correct asymptotic behavior (see Figure 4). Thus, assuming that CAM-B3LYP provides reasonable structures along these scans, the CASPT2 potential energy profiles for the T_1_ state shown in Figure 4b,d,f provide a reliable representation of the minimum energy paths for the photodissociation in the lowest triplet excited state from ^3^GS, ^3^MS2 and ^3^MS1, respectively. Note also that the relaxed pentacoordinated complex presents a *C*_2_ symmetry resulting from Jahn-Teller effects, that break the *C*_4_ symmetry of the doubly-degenerate *E* state.

**Figure 3:**
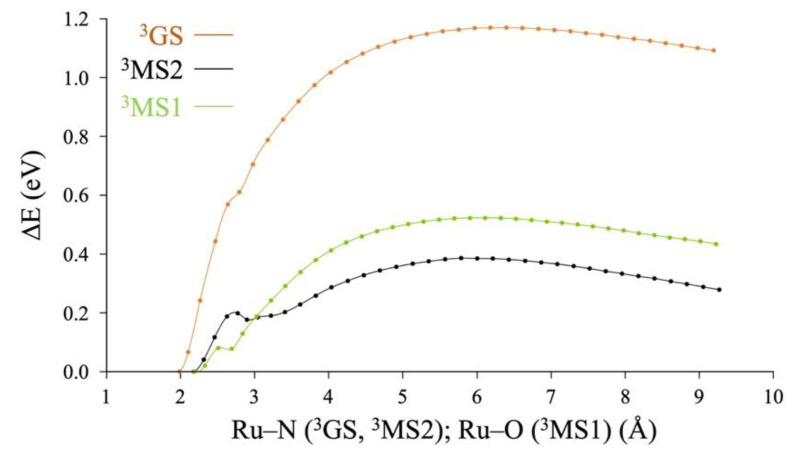
Minimum energy paths for NO dissociation in the *trans*-[RuCl(NO)(py)_4_]^2+^ complex at the B3LYP-D3 level of theory from the three triplet excited states ^3^GS, ^3^MS2 and ^3^MS1. The zero energy is set to the triplet energy of the ^3^GS, ^3^MS2 and ^3^MS1 at their respective minima for each curve. Adapted with permission from Ref. [50].

**Figure 4:**
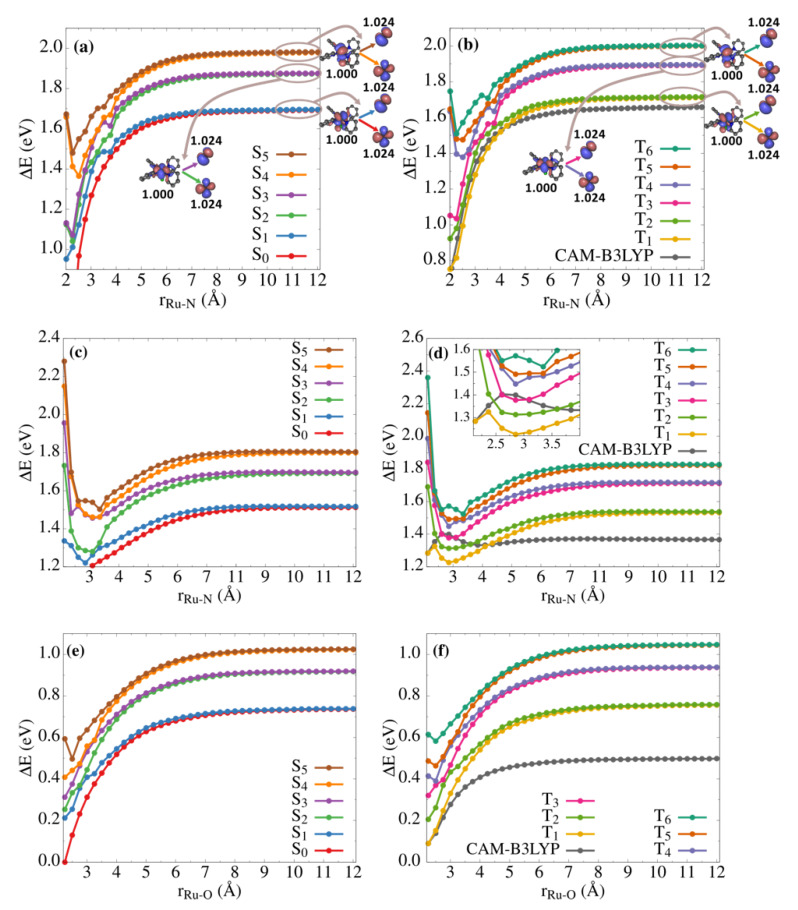
CASPT2 potential energy profiles along the Ru–NO or Ru–ON photodissociation for singlet states (**a**,**c**,**e**) and triplet states (**b**,**d**,**f**). Top, middle and bottom panels refer to photodissociation from ^3^GS, ^3^MS2 and ^3^MS1, respectively. The zero energy is set to the S_0_ electronic state. The CAM-B3LYP result is shown in (**b**,**d**,**f**) for each isomer. The hole/particle CASSCF molecular orbitals are also shown for each pair of degenerate states at the asymptotic limit along with their state-specific occupation numbers indicated in bold.

The photodissociation from ^3^GS shown in Figure 4b illustrates that there is a fairly good agreement between the CAM-B3LYP and CASPT2 potential energy profiles with dissociation energies of 0.91 eV and 0.96 eV, respectively, and smooth dissociation curves (weak state coupling). In contrast, for ^3^MS2 and ^3^MS1 (Figure 4d,f), the situation is very different with substantial deviations between the CAM-B3LYP and CASPT2 curves. While CAM-B3LYP predicts dissociation energies of 0.08 eV and 0.41 eV, respectively, the corresponding values at the CASPT2 level are 0.25 eV and 0.67 eV. In addition, Figure 4d shows a complex picture for the photodissociation curves from ^3^MS2 with many crossings occurring between the lowest triplet states including T_1_. In particular, a local potential energy well is created on the T_1_ potential energy surface at a Ru–N distance of about 2.8 Å due to a T_1_/T_2_ crossing. It is worth noting that this minimum is also visible at the B3LYP-D3 level (Figure 3) and at the CAM-B3LYP level (Figure 4d), and is also associated with a change of the electronic configuration in the unrestricted Kohn-Sham solution. At the CASPT2 level, this secondary minimum even appears lower in energy than that of ^3^MS2, which would need to be confirmed by geometry optimizations at the CASPT2 level beyond our available computational resources for such a large system. 

Overall, it is encouraging that, while the energetics are quantitatively different between the DFT and CASPT2 methods, the qualitative behavior between the two approaches is similar and the intermediate triplet species predicted to have the lowest Ru–NO dissociation energy is ^3^MS2, in agreement with the previous DFT study [50]. The dissociation energy curves on the singlet states are provided in Figure 4a,c,d for the sake of comparison, although photodissociation from the singlet excited states is less likely considering the efficiency of ISC in this type of systems after initial irradiation [45,49,53]. Note also that the lowest-energy dissociation path on S_0_ would follow a very different pathway from the ones shown in Figure 4, as it would involve dissociation to [Ru^II^Cl^−^(py)_4_]^+^ + NO^+^.

## 3. Computational Details 

The CASPT2 approach was used to describe the potential energy surfaces of the singlet ground state and lowest triplet excited states along the Ru–NO photodissociation pathways. Because of the issues described above with the B3LYP-D3 energy profiles, we have used the geometries coming out from relaxed scans along the Ru–N or Ru–O bond distances using the CAM-B3LYP functional [55]. The scans were started from ^3^GS, ^3^MS2 and ^3^MS1 structures optimized with CAM-B3LYP and the def2-TZVP basis set [56] on all atoms and the associated small-core relativistic pseudopotential for the Ru center [57]. These scans were performed at the same level of theory stepping along the Ru–N distance from ^3^GS and ^3^MS2, and along the Ru–O distance from ^3^MS1. The orientation of the departing NO was constrained by freezing some relevant angles during the scan.

**Figure 5:**
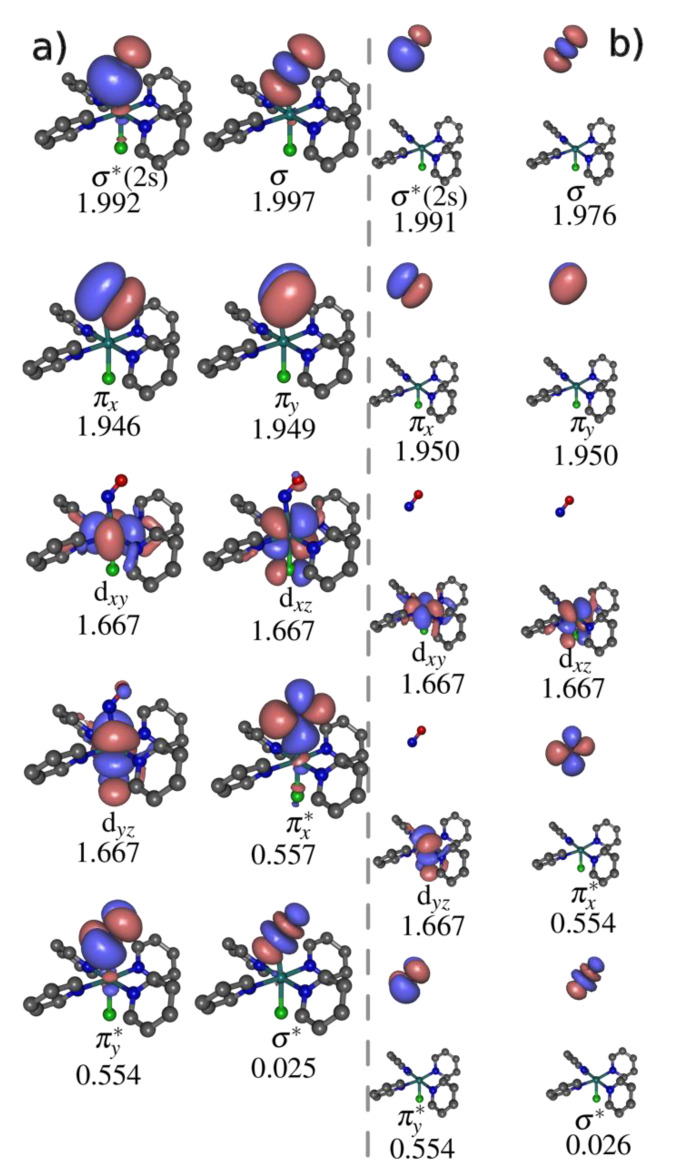
SA6-CASSCF(14,10) active space orbitals for the ^3^GS isomer, and their respective average occupation numbers for the triplet states (**a**) at the ^3^GS minimum and (**b**) at the asymptotic region.

The active space chosen for the underlying reference CASSCF calculations is shown in Figure 5 for the ^3^GS configuration and at its dissociation limit. A distribution of 14 electrons within 10 orbitals (i.e., CASSCF(14,10)) has been employed. The active space (active orbitals) is constituted of the main orbitals involved to describe the photodissociation process. The three occupied Ru(4d) valence orbitals correlating with the *t*_2*g*_ orbitals were included. The two *e*_g_ orbitals (d_z2_ and d_x2−y2_) does not appear to be involved in the NO dissociation. Two pairs of π and π* and the pair of σ and σ* orbitals of NO were also included, plus a σ*(2s). More extended active spaces have been tested, notably including the Ru(4d_x2−y2_) orbital and N(2p) orbitals of the equatorial pyridine ligands. Nevertheless, the CASSCF(14,10) proved to be the most stable with the proper asymptotic degeneracy expected for three consecutive pairs of singlet and triplet excited states (vide supra). Note that in linear configurations (e.g., like in GS and MS1), the 4d_z2_ also overlaps with the σ orbital [45], which does not appear to be the case when the Ru–N–O is bent (e.g., like in ^3^GS, ^3^MS2 and ^3^MS1).

The CASSCF calculations were performed averaging over 6 states for each spin state (singlet and triplet), in order to obtain good asymptotic behavior (degeneracies of pairs of electronic states due to the doubly-degenerate X^2^Π state of NO^•^) of the potential energy surfaces at the dissociation limit. For the same reason, multi-state CASPT2 (MS-CASPT2) was executed over 6 states. However, the MS-CASPT2 calculations proved to be extremely unstable at the asymptotic region because of artificial state mixing breaking the degeneracy of the doubly-degenerate X^2^Π state of NO^•^. This is a well-known issue of MS-CASPT2, which can occur when the electronic states are nearly or exactly degenerate [58]. Accordingly, only the state-specific CASPT2 (simply labelled as CASPT2) excited-state energies are reported in this study.

All the CASSCF and CASPT2 calculations were performed with the OpenMolcas program package [59], using the Cholesky decomposition algorithm with a threshold of 10^−6^ a.u. and the ANO-RCC-VTZP all-electron basis for Ru [60] and ANO-RCC-VDZP for all other atoms [61]. This precision in the two-electron integrals was required to solve degeneracy issues at the dissociation asymptotic region. A level shift of 0.3 a.u. and a standard IPEA shift [62,63] of 0.25 a.u. were used to run the CASPT2 calculations. The DFT calculations were performed with Gaussian 09 [64].

## 4. Conclusions

We report, in this work, accurate CASPT2 potential energy profiles of the lowest six singlet and six triplet states along the most probable photodissociation pathways of NO^•^ in the prototype *trans*-[RuCl(NO)(py)_4_]^2+^ complex. These calculations were performed from the three lowest triplet excited state intermediates previously identified in DFT studies [43,44,50]. By choosing an appropriate active space and treating several pairs of singlet and triplet states simultaneously, we address correctly the bond dissociation process and the degeneracies between the electronic states at the asymptotic limit.

Our results support the conclusion of a previous DFT study [50], which predicted the ^3^MS2 intermediate to be the most likely candidate for NO^•^ photorelease. Further, we report a dissociation energy from the triplet ^3^MS2 intermediate of only 0.25 eV, which is 0.71 eV and 0.42 eV lower than that from ^3^GS and ^3^MS1, respectively. Because population of this intermediate requires absorption of a second photon from MS2, the proposed mechanism for photorelease is that it is coupled to partial photoisomerization through a sequential two-step photon absorption mechanism. Because ^3^MS2 is also a key excited-state intermediate in the N→O linkage photoisomerization mechanism [43,44,45] leading to MS1, this triplet side-on species is possibly the branching structure on the lowest triplet potential energy surface for the two competing photochemical pathways: N→O linkage photoisomerization and NO^•^ photorelease.
